# BRASH Syndrome Unmasked: Digoxin Toxicity in the Setting of Cardiogenic Shock and Multiorgan Dysfunction

**DOI:** 10.1155/crcc/8778210

**Published:** 2026-07-15

**Authors:** Spencer Boehm, Jordan Bruni

**Affiliations:** ^1^ Department of Internal Medicine, Corewell Health Lakeland, Saint Joseph, Michigan, USA; ^2^ Department of Pharmacy, Corewell Health Lakeland, Saint Joseph, Michigan, USA

## Abstract

Digoxin, a cardiac glycoside historically used for rate control in atrial arrhythmias and symptom relief in chronic heart failure, possesses a narrow therapeutic index and is highly susceptible to pharmacokinetic and pharmacodynamic disruption in critically ill patients. Its use in acute decompensated heart failure and cardiogenic shock is therefore controversial. We present the case of a 75‐year‐old woman with chronic heart failure with reduced ejection fraction who developed progressive bradycardia and cardiogenic shock after treatment with digoxin and amiodarone for 2:1 atrial flutter. Her hospital course was complicated by acute kidney injury, severe hyperkalemia, ischemic hepatitis, and persistent bradycardia following electrical cardioversion. Serum digoxin levels were supratherapeutic early in the intensive care unit course and subsequently normalized, though bradyarrhythmias persisted. The patient′s presentation and clinical trajectory were consistent with BRASH (bradycardia, renal failure, AV‐nodal blockade, shock, and hyperkalemia) syndrome , with amiodarone–digoxin interaction and systemic hypoperfusion likely amplifying myocardial digoxin sensitivity. She improved with supportive therapy, correction of metabolic derangements, withdrawal of AV‐nodal‐blocking agents, and cardioversion, without administration of digoxin‐specific antibody fragments. This case highlights the importance of early recognition of BRASH physiology, cautious use of AV‐nodal blockers in low‐output states, and prioritization of underlying physiologic correction over reliance on serum drug levels alone in critically ill patients.

## 1. Introduction

Digoxin inhibits the Na^+^/K^+^–ATPase pump, increasing intracellular calcium and myocardial contractility while exerting vagomimetic effects at the atrioventricular (AV) node [1]. For decades, it has been used for ventricular rate control in atrial fibrillation and flutter and for symptomatic management of chronic heart failure [1–4]. However, its clinical utility in acute decompensated heart failure and cardiogenic shock is limited by delayed onset of action, reliance on renal clearance, and a narrow therapeutic window.

Contemporary heart failure guidelines discourage the routine use of digoxin in acute decompensation and cardiogenic shock due to its unpredictable pharmacokinetics in low‐output states and increased risk of toxicity in patients with renal dysfunction [5]. Additionally, digoxin is highly susceptible to clinically significant drug–drug interactions, particularly with amiodarone, which inhibits P‐glycoprotein–mediated digoxin clearance and can substantially increase serum and tissue exposure [6].

An increasingly recognized clinical entity, BRASH (bradycardia, renal failure, AV‐nodal blockade, shock, and hyperkalemia) [7–10] syndrome, describes a synergistic interplay between BRASH. In this syndrome, modest derangements in potassium or therapeutic‐range AV‐nodal blocker exposure can precipitate profound bradycardia and hemodynamic collapse. We present a case of digoxin toxicity occurring within this framework, illustrating the dangers of AV‐nodal blockade in the setting of evolving cardiogenic shock and multiorgan dysfunction.

## 2. Patient Consent for Publication

No formal written consent was obtained from the patient as there is no patient identifiable data included in this case report. The clinical course was reviewed retrospectively.

## 3. Case Presentation

### 3.1. Patient Information

A 75‐year‐old woman with a history of chronic heart failure with reduced ejection fraction, paroxysmal atrial fibrillation, severe pulmonary hypertension, and essential hypertension presented to the emergency department with progressive dyspnea and bilateral lower extremity edema. Her baseline functional status was independent and ambulatory living at home with her husband, body weight was 65.8 kg, home medications at the time included metoprolol 25 mg, apixaban 5 mg, levothyroxine 25 mg, sacubitril‐valsartan 24–26 mg, and furosemide 40 mg. Her most recent outpatient echocardiographic findings from a month prior to the current visit showed HFrEF with an ejection fraction of 35%. Her complete blood count on arrival, October 7, 2025, was unremarkable; her complete metabolic panel is provided in Table 1.

**Table 1:** Initial complete metabolic panel.

Test	Result	Reference range
Sodium	137 mmol/L	136–145
Potassium	3.7 mmol/L	3.4–5.0
Chloride	103 mmol/L	98–107
Bicarbonate (CO_2_)	21 mmol/L	22–29
Anion gap	13 mmol/L	5–14
Glucose	116 mg/dL	70–99
Blood urea nitrogen (BUN)	20 mg/dL	8–23
Creatinine	0.96 mg/dL	0.50–1.00
eGFR	62 mL/min/1.73 m^2^	> 60
Calcium	9.0 mg/dL	8.6–10.4
Total protein	6.5 g/dL	6.4–8.3
Albumin	3.5 g/dL	3.5–5.2
Globulin	3.0 g/dL	2.0–3.9
Albumin/globulin ratio	1.2	1.2–2.2
Total bilirubin	**1.9 mg/dL**	0.2–1.3
Alkaline phosphatase	**116 U/L**	35–104
ALT (SGPT)	27 U/L	0–35
AST (SGOT)	**41 U/L**	0–35

*Note:* Bold text reflects deviation from reference ranges.

### 3.2. Initial Clinical Findings and Emergency Department Course

**Figure 1:**
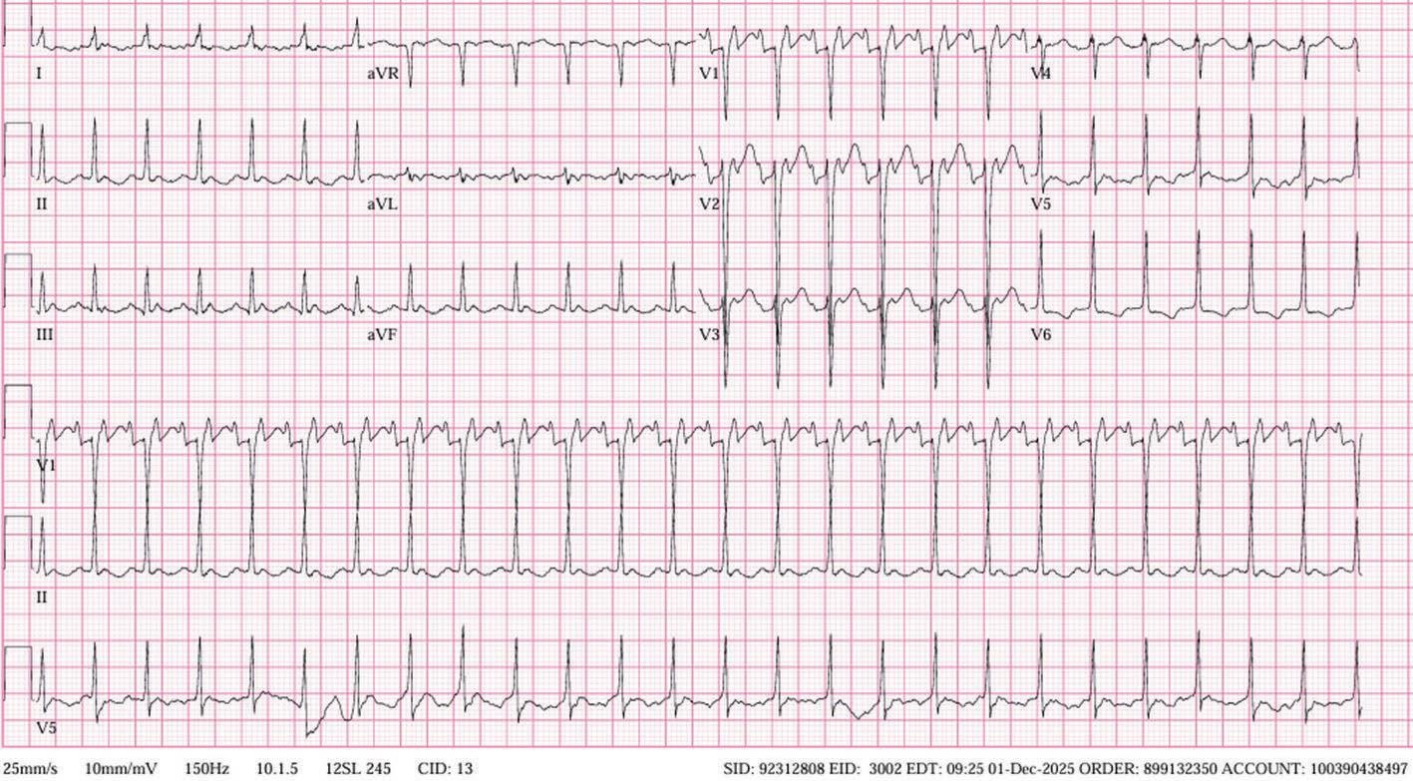
Initial electrocardiogram demonstrating atrial flutter with 2:1 conduction.

On presentation, the patient was tachycardic with a heart rate of approximately 150 beats per minute and was found to be in atrial flutter with 2:1 AV conduction. EKG is pictured in Figure 1.

She was hemodynamically stable at that time; however, she demonstrated a narrowed pulse pressure of 130/105 mmHg. Given the narrow pulse pressure and decreased LVEF, the emergency department elected to treat her tachycardia with amiodarone. She was also administered 40 mg of IV furosemide. Approximately 1 h later, the tachycardia persisted, prompting administration of 250 mcg of digoxin. Cardiology was consulted 1 h after the initial dose and recommended an additional 250 mcg of digoxin for heart rate control, as her heart rate remained at 136 bpm, which was promptly given. They further advised that if the heart rate did not improve to approximately 100 bpm, an additional 250 mcg of digoxin could be given after several hours, which was not administered prior to her admission. If these measures failed to adequately control her heart rate, the next recommended step was initiation of a diltiazem infusion, and she was subsequently admitted to the hospital for further management.

### 3.3. Hospital Course and Clinical Deterioration

The patient was admitted to the medical floor, and the amiodarone infusion was continued. Metoprolol therapy was also initiated and titrated, and diuresis was intensified. Digoxin was not continued beyond the initial emergency department doses. Initial laboratory evaluation is shown in the following table, significant for mild hypokalemia, which was subsequently repleted, as well as an acute kidney injury. Repeat CMP on admission is shown in Table 2.

**Table 2:** CMP obtained by the admitting hospitalist team.

Test	Result	Reference range
Sodium	139 mmol/L	136–145
Potassium	**3.3 mmol/L**	3.4–5.0
Chloride	103 mmol/L	98–107
Bicarbonate (CO_2_)	24 mmol/L	22–29
Anion gap	12 mmol/L	5–14
Glucose	107 mg/dL	70–99
Blood urea nitrogen (BUN)	**28 mg/dL**	8–23
Creatinine	**1.96 mg/dL**	0.50–1.00
Calcium	9.6 mg/dL	8.6‐10.4

*Note:* Bold text reflects deviation from reference ranges significant to the case.

Within the first 24 h of hospitalization, the patient developed progressive respiratory distress and hypotension consistent with evolving biventricular cardiogenic shock. Milrinone was initiated for inotropic support; however, she subsequently developed worsening hypotension, hypothermia, and altered mental status. Laboratory studies demonstrated acute kidney injury, severe hyperkalemia, high anion gap metabolic acidosis, hyperglycemia, and marked transaminase elevation consistent with ischemic hepatitis, prompting intensive care consultation. The initial labs drawn in the ICU on October 8, 2025, are shown in Table 3.

**Table 3:** Complete metabolic panel obtained upon transfer to the intensive care unit.

Test	Result	Reference range
Sodium	139 mmol/L	136–145
Potassium	**7.1 mmol/L**	3.4–5.0
Chloride	100 mmol/L	98–107
Bicarbonate (CO₂)	12 mmol/L	22–29
Anion gap	26 mmol/L	5–14
Glucose	261 mg/dL	70–99
Blood urea nitrogen (BUN)	29 mg/dL	8–23
Creatinine	**2.09 mg/dL**	0.50–1.00
eGFR	24 mL/min/1.73 m^2^	> 60
Calcium	9.0 mg/dL	8.6–10.4
Total protein	5.9 g/dL	6.4–8.3
Albumin	3.1 g/dL	3.5–5.2
Globulin	2.8 g/dL	2.0–3.9
Albumin/globulin ratio	1.1	1.2–2.2
Total bilirubin	**1.8 mg/dL**	0.2–1.3
Alkaline phosphatase	105 U/L	35–104
ALT (SGPT)	**1126 U/L**	0–35
AST (SGOT)	**2566 U/L**	0–35

*Note:* Bold text reflects deviation from reference ranges pertinent to the case.

### 3.4. Intensive Care Unit Course

The patient was transferred to the intensive care unit, where vasopressor and inotropic support were initiated with norepinephrine and dobutamine. Amiodarone and milrinone were discontinued due to worsening hypotension and concern for exacerbation of bradycardia. Hyperkalemia and acidemia were treated with insulin–dextrose therapy, sodium bicarbonate infusion, potassium‐binding agents, and inhaled *β*‐agonists. Due to clinical concern for intravascular depletion despite reduced ejection fraction, a cautious crystalloid bolus was administered.

**Figure 2:**
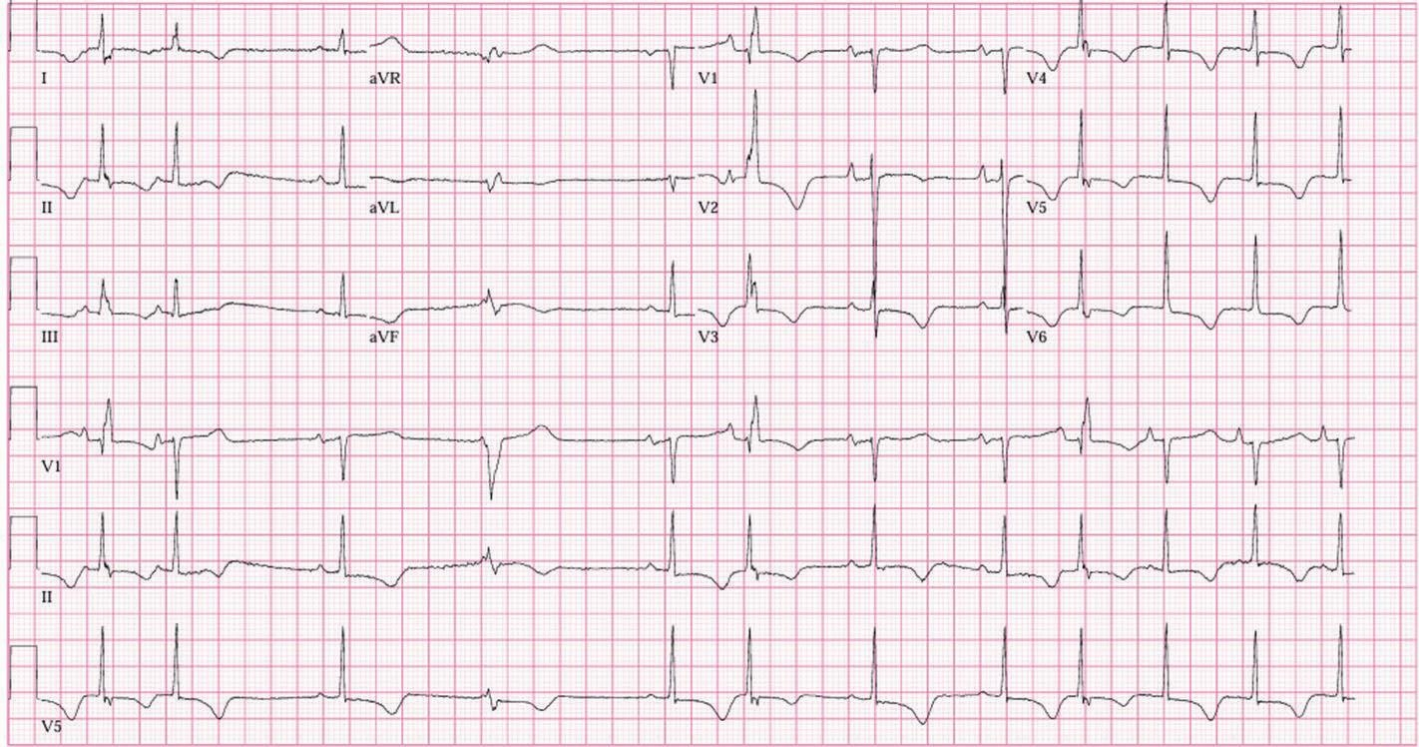
Postcardioversion electrocardiogram demonstrating restoration of sinus rhythm.

A serum digoxin level obtained during the intensive care unit course was elevated at 2.2 ng/mL. Despite correction of metabolic derangements and hemodynamic stabilization, the patient demonstrated intermittent bradycardia with an irregular rate that would quickly convert to rate controlled atrial fibrillation. After clinical stabilization, transesophageal echocardiography‐guided electrical cardioversion was performed, successfully restoring sinus rhythm with occasional premature ventricular contractions. Post cardioversion EKG is provided below; however, intermittent bradycardic periods with rates as low as 38 bpm persisted intermittently over the next 24 h and were noted during observation on telemetry (Figure 2).

No timestamped telemetry events of these bradycardic periods could be obtained. Her blood pressure was otherwise stable during these events, and she remained asymptomatic despite these findings. A limitation of this case is that no saved telemetry strip or timestamped ECG capture was available to document the intermittent bradycardic episodes observed after cardioversion. Therefore, the precise rhythm mechanism during these episodes could not be definitively characterized, though it appeared to be a junctional bradycardia that spanned from seconds to a minute that would subsequently revert to a normal sinus rhythm.

## 4. Outcome and Follow‐Up

**Figure 3:**
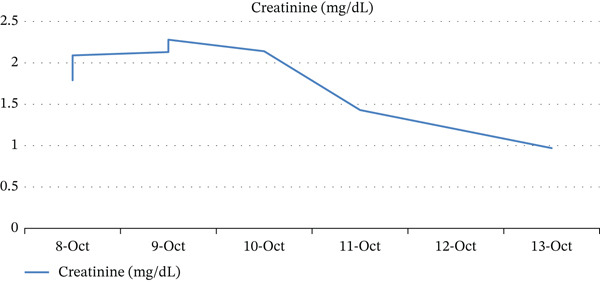
Trend of serum creatinine during the ICU course.

**Figure 4:**
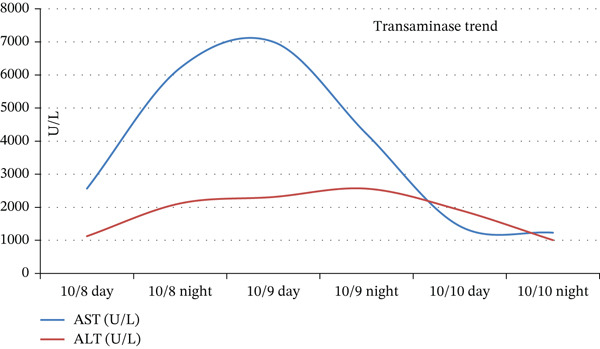
Trend of serum transaminases during the ICU course.

Over the subsequent 48 h, the patient′s hemodynamic status improved, and vasopressor support was weaned with eventual discontinuation, renal function stabilized, and liver enzymes began to quickly downtrend. Potassium remained stable; but the overall trend of serum transaminases and serum creatinine is illustrated in the following graph within the ICU clinical course (Figures 3 and 4).

**Figure 5:**
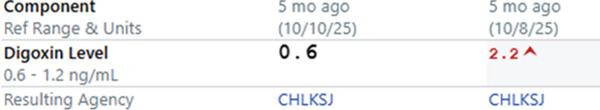
Serum digoxin level trend during hospitalization.

Serum digoxin levels stabilized with supportive care alone. Demonstration of the repeat level is provided in Figure 5.

The bradycardia gradually resolved without need for pacing or digoxin‐specific antibody fragments. The patient was transferred back to the general medical floor and later discharged in stable condition, and labs returned to normal prior to discharge on October 13, 2025, with only minor residual increases in transaminases and a slightly elevated serum creatinine.

## 5. Discussion

This case illustrates the dangers of digoxin use in the setting of evolving cardiogenic shock and multiorgan dysfunction and is best interpreted through the framework of BRASH syndrome. The patient′s clinical course—progressive bradycardia, acute kidney injury, severe hyperkalemia, concurrent AV‐nodal‐blocking agents, and hemodynamic collapse—fits squarely within this increasingly recognized pathophysiologic entity. The patient′s clinical course was consistent with BRASH physiology, which provides an important framework for understanding the synergistic interaction among renal dysfunction, hyperkalemia, AV‐nodal blockade, and shock. However, the presentation was likely multifactorial, with possible contributions from evolving cardiogenic shock, amiodarone–digoxin interaction, and digoxin‐related conduction effects.

BRASH syndrome represents a synergistic, self‐perpetuating cycle in which renal hypoperfusion leads to accumulation of AV‐nodal blockers and potassium, whereas hyperkalemia and AV‐nodal blockade compound one another to produce profound bradycardia and reduced cardiac output. This further worsens renal perfusion, accelerating drug accumulation and potassium retention. Importantly, bradycardia in BRASH syndrome may be disproportionate to the degree of hyperkalemia or serum drug levels, reflecting a multiplicative rather than additive electrophysiologic effect [7–10]. Early recognition of this physiology is critical, as standard reflexive escalation of AV‐nodal blockade or delayed cardioversion can exacerbate the underlying vicious cycle.

In this case, the patient received multiple AV‐nodal‐modulating agents—digoxin, amiodarone, and metoprolol—in the context of rapidly worsening renal function. Digoxin is highly susceptible to accumulation in low‐output states due to its dependence on renal clearance and extensive tissue distribution. Although the measured digoxin concentration was only modestly elevated, serum levels do not reliably reflect myocardial tissue burden in critically ill patients. Digoxin′s large volume of distribution (Vd) and slow redistribution mean that clinical toxicity may persist despite normalization of serum concentrations, as evidenced by this patient′s persistent bradycardia following correction of metabolic derangements and cardioversion.

The interaction between amiodarone and digoxin further amplified this risk. Amiodarone inhibits P‐glycoprotein–mediated digoxin clearance, predictably increasing serum concentrations and enhancing myocardial sensitivity even without further dosing [6]. This interaction can occur rapidly and may be magnified in the presence of renal dysfunction. Importantly, the pharmacodynamic consequences may outlast detectable serum elevation, reinforcing the need to prioritize clinical status over laboratory thresholds alone when assessing toxicity.

Beyond renal dysfunction, this patient developed marked transaminase elevation consistent with ischemic hepatitis secondary to cardiogenic shock, an often‐underappreciated modifier of drug effect. Hepatic hypoperfusion may alter plasma protein binding, reduce first‐pass metabolism of coadministered drugs, and shift the balance toward higher free (pharmacologically active) drug fractions. In critically ill patients with systemic hypoperfusion, even drugs that are not primarily hepatically cleared—including digoxin—may exhibit exaggerated pharmacodynamic effects due to altered distribution and receptor sensitivity. This hepatic contribution likely further increased the patient′s susceptibility to digoxin‐associated conduction disturbances. The expected bioavailability based on digoxin′s Vd of 5–10 L/kg, after a 500 mcg dose, was calculated and the range of serum levels would be projected to be 0.69–1.38 ng/mL with the sole administration occurring in the emergency department 12 h prior to her arriving at the intensive care unit, whereas our patient’s serum level was much higher at 2.2 ng/mL.

Management of BRASH physiology differs from isolated digoxin toxicity or simple hyperkalemia. The primary therapeutic priorities include immediate cessation of AV‐nodal‐blocking agents, aggressive correction of hyperkalemia and acidosis, and restoration of perfusion with appropriate vasopressor and inotropic support. Chronotropic support may be required temporarily, but definitive improvement depends on interrupting the reinforcing cycles of renal failure, drug accumulation, and electrolyte disturbance. Electrical cardioversion remains the most effective strategy for rhythm control in unstable patients and should not be delayed once metabolic stabilization is underway.

The role of digoxin immune Fab was carefully considered in this case. Although the patient exhibited significant clinical features consistent with digoxin toxicity, Fab therapy was deferred due to stabilization with supportive care, resolution of hyperkalemia, absence of malignant ventricular arrhythmias or high‐grade AV block, and improving hemodynamics. In chronic or subacute toxicity, particularly in the setting of BRASH syndrome, serum digoxin concentrations alone may not mandate Fab administration if clinical improvement is evident. Nevertheless, this case underscores the importance of maintaining a low threshold for Fab therapy if bradyarrhythmias or hemodynamic instability persist despite correction of metabolic abnormalities. Although the patient had severe hyperkalemia during the ICU course, this improved promptly with insulin–dextrose therapy, bicarbonate, *β*‐agonist therapy, potassium‐binding agents, and restoration of perfusion. In the absence of malignant ventricular arrhythmias, high‐grade AV block, persistent hemodynamic instability, or ongoing hyperkalemia after initial therapy, digoxin‐specific Fab was deferred with close monitoring. Fab therapy would have been reconsidered if bradyarrhythmia, hyperkalemia, or shock had persisted despite correction of the underlying BRASH physiology.

This case highlights several important lessons. First, BRASH syndrome should be actively considered in patients presenting with bradycardia and shock in the setting of renal dysfunction and AV‐nodal blocker exposure, even when hyperkalemia or drug levels appear only modestly abnormal. Second, digoxin′s narrow therapeutic index and delayed tissue kinetics make it a particularly risky agent in evolving shock states. Third, hepatic hypoperfusion may significantly modify drug effects during cardiogenic shock and should be considered when evaluating unexplained toxicity. Finally, management should prioritize early recognition, rapid withdrawal of precipitating agents, and targeted correction of the underlying physiology rather than reliance on drug levels alone, while maintaining a low threshold to consider digoxin‐specific antibody fragments (Digibind/DigiFab) in the presence of persistent bradyarrhythmias, refractory hyperkalemia, or ongoing hemodynamic instability despite initial supportive therapy.

## 6. Conclusion

This case demonstrates the dangers of digoxin use in patients with evolving cardiogenic shock and emphasizes the importance of recognizing BRASH syndrome as a unifying pathophysiologic framework for unexplained bradycardia and hemodynamic collapse. In such settings, even standard doses of AV‐nodal‐blocking agents may precipitate severe toxicity due to renal dysfunction, electrolyte disturbances, impaired hepatic perfusion, and heightened myocardial sensitivity.

Serum digoxin concentrations may underestimate true pharmacologic effect in critically ill patients, and persistent conduction abnormalities may reflect delayed tissue clearance rather than continued exposure. Early identification of BRASH physiology, prompt withdrawal of AV‐nodal blockers, aggressive correction of hyperkalemia, and restoration of systemic perfusion are central to successful management. This case underscores the need for cautious pharmacologic decision‐making in unstable patients and highlights the educational value of applying emerging clinical frameworks to complex drug‐toxicity presentations.

## Funding

No funding was received for this manuscript.

## Conflicts of Interest

The authors declare no conflicts of interest.

## Data Availability

The data that support the findings of this study are available from the corresponding author upon reasonable request.
